# Vitamin D Binding Protein Gene Polymorphisms (rs4588 and rs7041) and VDBP Levels in Total Hip Replacement Outcomes

**DOI:** 10.3390/nu17030378

**Published:** 2025-01-21

**Authors:** Dominika Rozmus, Ewa Fiedorowicz, Janusz Płomiński, Anna Cieślińska

**Affiliations:** 1 Department of Biochemistry, Faculty of Biology and Biotechnology, University of Warmia and Mazury in Olsztyn, 10-719 Olsztyn, Poland; ewa.kuzbida@uwm.edu.pl (E.F.); anna.cieslinska@uwm.edu.pl (A.C.); 2 Orthopaedic and Trauma Teaching Hospital, 05-400 Otwock, Poland; plominsky@poczta.onet.pl

**Keywords:** vitamin D binding protein, vitamin D, 25(OH)D, rs7041, rs4588, total hip replacement, nutrigenomics

## Abstract

**Background:** Total hip replacement (THR) significantly improves patients’ quality of life; however, prosthesis loosening remains a significant complication. Vitamin D, essential for calcium homeostasis and bone mineralization, is transported and stabilized by vitamin D binding protein (VDBP). Common single nucleotide polymorphisms (SNPs) in the VDBP gene, rs4588 and rs7041, may influence serum vitamin D levels and potentially impact THR outcomes. This study aimed to analyze the association between these SNPs, serum levels of VDBP and 25(OH)D, and their potential roles in THR outcomes. **Methods:** The study included three patient groups: (1) patients undergoing arthroscopy after a THR without prosthesis loosening (CA—Control Arthroplasty), (2) patients with hip prosthesis loosening (L—Loosening), and (3) a control group (C—Control). Genotyping of rs4588 and rs7041 in the VDBP gene was conducted using PCR-RFLP and TaqMan Genotyping real-time PCR. Serum levels of VDBP and 25(OH)D were measured using ELISA. Comparisons between groups were performed using statistical analyses, including odds ratios (OR) and significance testing (*p*-values). **Results:** There are significant differences in VDBP concentrations between the groups: L vs. CA (*p* < 0.0001), L vs. C (*p* = 0.0118), L vs. L + CA (*p* = 0.0013), CA vs. C (*p* < 0.0001), and CA vs. L + CA (*p* < 0.0001), and in 25(OH)D concentrations between groups: L vs. C (*p* < 0.0001), CA vs. C (*p* = 0.0008), and C vs. L + CA (*p* < 0.0001). **Conclusions:** The study findings suggest a protective role of 25(OH)D against prosthesis loosening in THR. The rs4588 SNP in the VDBP gene may increase the risk of loosening, while differences in VDBP and 25(OH)D concentrations between patient groups highlight their potential importance in THR outcomes.

## 1. Introduction

Total hip replacement (THR)/Total Hip Arthroplasty (THA) is a standard procedure for the treatment of end-stage hip osteoarthritis, providing pain relief and improved joint function. The available statistical data show that the main causes of prosthesis loosening are inflammation: septic (7.5%), aseptic (55.2%), and dislocations and broken prostheses (18.8%)—this group also includes surgical errors that constitute 3.8% and events not mentioned in the statistics [1,2]. In the case of failed THRs, revision total hip replacement is a salvage procedure with increased surgical risks, including sepsis, infection of the prosthetic joint, prolonged operative time and anesthetic exposure, increased blood loss, higher treatment costs, and longer hospital stay [3].

According to the statistics, Switzerland is the country with the highest number of THRs performed (322.6 procedures on 100,000 citizens). In the range of 250–300 procedures per 100,000 citizens are performed also in Germany, Austria, Finland, Belgium, Norway, and the Netherlands. In the range of 200–249 are performed in France, Denmark, Iceland, Iceland, Sweden, and Luxembourg. A total of 183.1 procedures have been formed in Poland, while Canada has performed 152.7, Israel 73, Korea 64.2, and Mexico 6.9 per 100,000 citizens, presenting the lowest number of surgeries “www.statista.com (accessed on 3 January 2025)” [4]. According to the American Association of Hip and Knee Surgeons, the data suggest that hip replacements have an annual failure rate in the range of 0.5–1.0% [5].

The data indicate that, in Poland in 2024, 64,140 total hip replacements were performed, and these accounted for 57.4% of all endoprosthetic procedures. Primary THRs constituted 92.6% of all THRs, while revision THRs—7.4% [6].

The Swedish arthroplasty register showed that, in 2022, 20,568 primary hip replacements were registered [7]. The national report of Norway states 9396 primary operations, 112 reoperations, and 1172 revisions in 2021, which presents a total of total 10,680 surgeries [8]. The report from the UK shows 108,558 primary THRs and 6659 revision THRs were performed in 2023 [9].

The role of vitamin D, an important element of the endocrine system, in bone health is well documented. In addition, one of the problems associated with vitamin D metabolism is the loosening of hip prostheses [10], which is best defined as migration caused by poor locking, poor bone quality, or premature bone resorption [11]. The action of vitamin D (VD) influences the proper function of many organs in the body [12], and the best known role of its active form (1,25-dihydroxyvitamin D; 1,25(OH)_2_D) is the regulation of calcium and phosphorus metabolism, and the maintenance of the proper condition of the skeletal system [13,14].

Vitamin D circulating in the human body requires a transporter, a serum alpha2-globulin called the vitamin D binding protein (*VDBP*) [15,16], or originally a “group specific component”—Gc [17]. The *VDBP* is produced in the liver by hepatic parenchymal cells [18] under the control of estrogen, glucocorticoids, and inflammatory cytokines, but not vitamin D itself [19]. Animal studies suggest that the *VDBP* prevents the degradation of 25(OH)D, which prolongs 25(OH)D half-life, and has a protective effect against vitamin D deficiency [20].

Vitamin D can enter the human body in two ways: it can be supplied by food [21] (vitamin D_2_ or D_3_ [22]) and absorbed via the intestine [21] or it can be produced in the skin (vitamin D_3_) during sun exposure under UVB radiation [23] (a wavelength of 290–315 nm [24], with the optimal peak at 295 nm) from 7-DHC [25]. These forms must be hydroxylated in the two most important organs, the liver and the kidneys, in order to become active. Therefore, they are transported by the *VDBP* to each of the organs where the inactive form is metabolized (hydroxylated) [24]. The first organ to metabolize vitamin D is the liver, where vitamin D is hydroxylated to 25(OH)D by CYP2R1 (25-hydroxylase). Subsequently, 25(OH)D is transported to the *VDBP* to other tissues, such as the kidney, where it is metabolized to 1,25(OH)_2_D by another enzyme—CYP27B1 (1-α-hydroxylase) [19]. There are also other enzymes involved in hydroxylation processes, such as the enzyme CYP11A1, which leads to the formation of vitamin D hydroxymetabolites that better protect the skin from DNA damage and oxidative stress. These metabolites promote keratinocyte differentiation; have anti-inflammatory, anti-fibrogenic, and anti-cancer properties; and inhibit cell proliferation in a structure-dependent manner [26,27].

**Figure 1:**
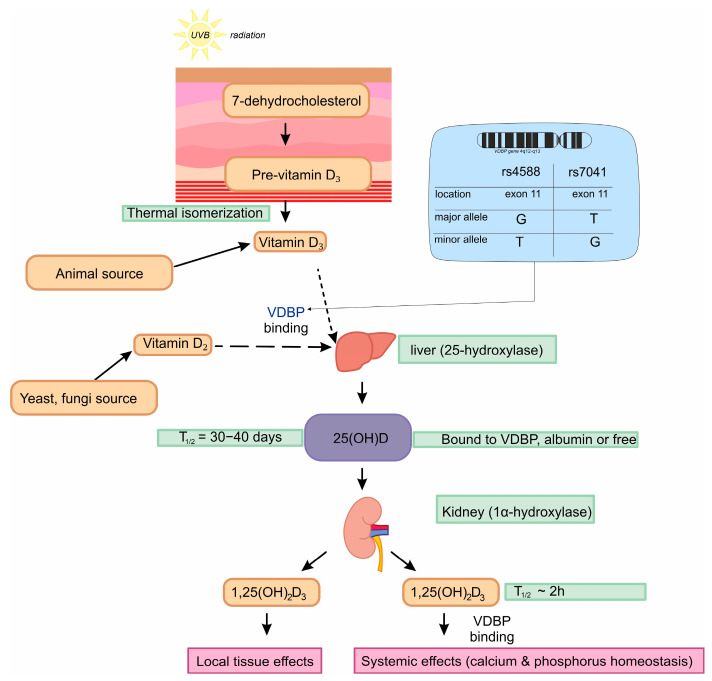
Metabolic pathway of vitamin D. Figure was made with SciPaitner beta; “www.scipainter.com (accesed on 20 January 2025)”.

The metabolic pathway of vitamin D is shown in Figure 1. All VD metabolites are transported by the VDPB to the target tissues, where the effect of VD is mediated by the VDR (Vitamin D Receptor), a nuclear receptor found in every cell of the body [18].

We previously examined the association of the VDR in prostheses loosening [28] among Total Hip Arthroplasty (THA)/THR patients, and the findings prompted us to expand our research to other factors concerning the metabolic pathway of vitamin D, like the vitamin D binding and transporter protein, which also might have an influence on prostheses loosening.

The *VDBP* gene is a 35 kb DNA consisting of 13 exons and 12 introns located on the long arm of chromosome 4 (4q12-q13) [29]. The *VDBP* is also known to bind extracellular actin and facilitate the transport of fatty acids. Additionally, it seems to safeguard the complement factor C5a from proteolytic degradation, thereby enhancing its function as a chemotactic protein [30]. The *VDBP* has a single binding site for all vitamin D metabolites, forming a large circulating pool of 25(OH)D that helps prevent the rapid onset of vitamin D deficiency [31]. The vitamin D binding properties of the *VDBP*, combined with its potential direct effects on bone resorption, have gained significant scientific interest regarding its role in bone metabolism and health. This interest is increased further by the discovery of notable interindividual variations in *VDBP* levels. The research findings suggest a connection between these variations and differences in bone density [32].

Rs7041 and rs4588 single nucleotide polymorphisms (SNPs) are located in exon 11 of the *VDBP* gene [33] and generate three major isoforms of the *VDBP*, including GC-1F, also known as a wild type, GC1S, and GC-2 [34] (Figure 1). These haplotypes are known to regulate the concentration of the *VDBP*, its affinity for 25 OH vitamin D, and thereby affect the levels of free vitamin D [35].

It has been found that rs4588 and rs7041 are associated with many diseases, such as tuberculosis, diabetes mellitus, obesity, chronic obstructive pulmonary disease, and others [36]. According to the NCBI frequency information, in rs4588 (G > T) among the European population, the most common allele is G (72%) [37], and in rs7041 (T > G) it is G (53%) [38].

The genetic variability in the *VDBP* gene and the role of vitamin D in bone remodeling raise the question of whether individual differences can influence the risk of hip prosthesis loosening. This study is of crucial importance in the context of hip replacement, as vitamin D deficiency as well as THRs are particularly common in older populations. The aim of this study is therefore to investigate the relationship between *VDBP* polymorphisms (rs4588 and rs7041), serum *VDBP* protein, and 25(OH)D levels in three groups of patients: (1) a group of arthroscopy patients after receiving THRs without prosthesis loosening, (2) patients after receiving THRs with a loosened hip prosthesis, and (3) the control group (healthy subjects).

This study’s objective was to investigate the relationship between the two most-common single nucleotide polymorphisms (SNPs) of the vitamin D binding protein (*VDBP*) gene (rs4588 and rs7041), the serum levels of *VDBP* and 25(OH)D, and their potential roles in prosthesis loosening following a total hip replacement (THR) in a case–control study. This study aims to explore whether these genetic and biochemical factors contribute to prosthesis stability and patient outcomes.

We hypothesize that genetic variations in the *VDBP* gene (rs4588 and rs7041) and differences in the serum levels of *VDBP* and 25(OH)D are associated with an increased risk of prosthesis loosening after a THR. Specifically, lower 25(OH)D levels and specific *VDBP* SNPs may influence the likelihood of this complication.

## 2. Materials and Methods

### 2.1. Characteristic of Patients and Control Groups

#### 2.1.1. Recruitment and Sample Collection

Patients were recruited for this case–control study between 2019 and 2024, and the samples were collected each year between November and March at the Voivodal Specialistic Hospital in Olsztyn, Poland, and the Gruca Orthopaedic and Trauma Teaching Hospital in Otwock, Poland. All the required data were collected from patients as medical records and/or a completed questionnaire. All participants provided informed consent for the study, which was in accordance with the ethical standards of the Declaration of Helsinki. This research was certified by the Bioethics Commissions (49/2019 and 139/2023).

The patients were divided into three groups: (1) a group of patients with a hip replacement in whom the transplant was rejected (L—Loosening); (2) a group of patients with a hip replacement in whom the transplant was functioning correctly and there was no loosening (CA—Control Arthroplasty); and (3) a group of healthy volunteers (C—Control). The study sample was carefully selected to focus on patients with specific characteristics of prosthetic loosening, ensuring homogeneity. The characteristics of groups, including age and gender, are shown in Table 1.

This case–control study followed the STROBE guidelines.

#### 2.1.2. Exclusion and Inclusion Criteria

Rigorous inclusion and exclusion criteria, based on clinical and radiographic assessments, were applied to maintain the integrity of the findings. In groups L and CA, the diagnosis was confirmed through clinical examinations and radiographs, and both groups had cementless prostheses. The primary symptoms of loosening included pain in the lower limb during weight bearing, pain in the groin or thigh, new popping or clicking sounds, joint instability, and the dislocation or subluxation (partial dislocation) of the joint. These symptoms appeared 3 to 12 years after the initial prosthetic surgery. Loosening was diagnosed based on specific radiographic criteria, including periprosthetic radiolucent lines wider than 2 mm or prosthetic migration greater than 4 mm. Exclusion criteria were patients with a fistula, those who experienced surgical errors, and those who did not meet the time criteria. Patients with a BMI over 35 were also excluded. Additionally, individuals with osteoporosis and rheumatic diseases were not included, as these conditions affect prosthesis survival. Therefore, patients with loosening due to fractures were not part of this study.

**Figure 2:**
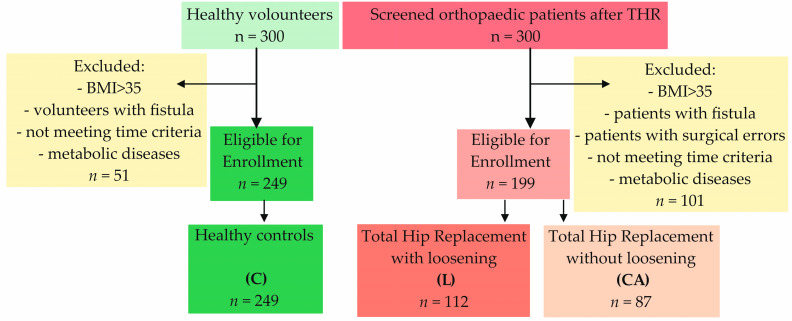
Flowchart of patient and volunteer enrollment.

Inclusion criteria for control group: healthy subjects with no orthopedic medical history. The flowchart of the patients’ enrollment is presented in Figure 2.

### 2.2. Biological Material Collection

Approximately 5 mL of peripheral blood and 5 mL of serum were collected from each participant in the seasons of late autumn and winter (November–March) in the years 2019–2023. Blood collection took place during a follow-up visit, during which prosthesis component loosening was confirmed based on clinical and radiological examinations. The samples were immediately transported to the laboratory, where they were either analyzed directly or stored at −80 °C for future use. DNA was extracted and purified using the Blood Mini DNA Isolation Kit and Sherlock AX (A&A Biotechnology, Gdańsk, Poland). The extracted DNA was diluted to a concentration of 20 ng/µL in preparation for genetic analysis and stored at −20 °C until further processing. Serum, which, due to its condition, was not suitable for testing, was rejected.

### 2.3. Concentration Assessment and Calculation

25(OH)D and the *VDBP* were assessed using ELISA assays, and the data were calculated and used as continuous variables in the statistical analysis.

#### 2.3.1. 25(OH)D Serum Concentration

Serum levels of 25(OH)D were determined using an ELISA assay (*n* = 199), performed in two runs, with a kit from Demeditec Diagnostics GmbH (Kiel, Germany). Statistical analysis was conducted using GraphPad Prism software. A standard curve was generated, and two controls were prepared by reconstituting the kit’s calibrators in 1 mL of distilled water. A 25(OH)D conjugate concentrate was diluted at a ratio of 1:100. The HRP conjugate was diluted at 1:200 and prepared approximately 2 h before use by combining the following reagents in order: conjugate buffer, concentrated buffer, vortexing, then adding concentrated HRP, and vortexing again. The wash solution was diluted with water at 1:200. In each appropriate well, 50 μL of the calibrator, control, and sample were added along with 150 μL of incubation buffer. The plate was then incubated for 2 h at room temperature on a plate shaker at 400 rpm. Following incubation, the liquid was aspirated, and the plate was washed three times with 350 μL of wash solution, with aspiration after each wash. Next, 200 μL of the working HRP conjugate solution was added to each well, and the plate was incubated again for 30 min at room temperature on the plate shaker (400 rpm). After this, the liquid was aspirated, and the plate was washed three more times. A chromogenic solution (100 μL) was added to each well, and the plate was incubated for 15 min at room temperature on the shaker at 400 rpm. Finally, 100 μL of stop solution was added to each well, and the absorbance was read at 450 nm with a 650 nm reference immediately after the addition of the stop solution.

#### 2.3.2. Vitamin D Binding Protein Concentration

The *VDBP* was measured (*n* = 284) using an ELISA assay: “Human Gc/*VDBP*” (Cat. No E1810h, EIAab Science Co., Wuhan, China). The procedure followed the manual added to the kit: standard curves were made by reconstituting the standards with 1 mL of sample diluent (7000 pg/mL) and making seven serial dilutions. The sample diluent served also as a zero standard (0 pg/mL). Reagents A and B were diluted to working concentrations of 1:100 with diluents A and B, respectively. Before pipetting, the reagents were mixed thoroughly. A total of 100 μL of standards, blanks, and samples was added to each well and covered with the plate sealer for the whole incubation time. Samples were incubated for 2 h at 37 °C. Detection reagent A working solutions of 100 µL were added to each well and covered with a plate sealer. The samples were incubated for another 1 h at 37 °C. Wells were aspirated and washed for a total of three times with washing buffer diluted with distilled water. After washing and the aspirating process, 100 µL of detection reagent B was added and covered with a plate sealer, and the samples were incubated for another 1 h at 37 °C. After incubation, the aspiration and washing processes were repeated. The substrate working solution in the amount of 100 µL was added to each well. After 20 min, the reaction was stopped using the stop solution and absorbance was measured at 450 nm with Asys UVM-340 (Biochrome, Cambridge, UK). Samples whose results were too high compared to the standard absorbance curve were measured again with the same kit in duplicates after dilution twice with the sample diluent. Statistical analysis was performed using GraphPad Prism 10.2.3 (GraphPad Software, Inc., Solana Beach, CA, USA).

### 2.4. Genotyping

#### 2.4.1. *rs7041*

The mixture for amplification at a volume of 17.5 μL consisted of the DreamTaq™ Green Master Mix (Thermo Scientific, Waltham, MA, USA); specific primers (F: 5-ctggcagagcgactaaaagc-3; R: 5-ccaggaaaagcctgtcacat-3; Sigma-Aldrich, Burlington, MA, USA) based on the studies of Harsini [39] and Wang [40], with their own modifications made using the online primer design tool (Primer3Plus, version: 3.3.0); and the DNA matrix and nuclease-free water (Thermo Scientific, Waltham, MA, USA) in the Mastercycler Nexus (Eppendorf, Hamburg, Germany). The steps employed were as follows: initialization at 95 °C for 5 min, and 35 cycles of denaturation at 95 °C for 60 s, annealing at 60 °C for 60 s, and elongation at 72 °C for 60 s. Afterwards, the last cycle mixture was incubated for 10 min at 72 °C for the final extension and then cooled at 4 °C before further analysis. The yield and specificity of the PCR product (248 bp) were evaluated by electrophoresis in 2.5% agarose gel (Promega, Fitchburg, WI, USA) and staining with GelGreen Nucleic Acid Gel Stain (Biotium, Fremont, CA, USA). The amplified fragments were digested with the restriction enzyme BsurRI (Thermo Scientific, Waltham, MA, USA), according to the manufacturer’s instructions, and visualized on 2.5% agarose gel (TT: 248 bp; TG: 248, 220, 28 bp; GG: 220, 28 bp). Sample size *n* = 448. The DNA sequencing of randomly selected samples after amplification was performed to confirm correct genotyping by an external company.

#### 2.4.2. *rs4588*

The mixture for genotyping was prepared in a volume of 10 μL per reaction and consisted of 5.0 μL of a TaqMan™ Universal PCR Master Mix (ThermoFisher Scientific, Waltham, MA, USA), 4.0 μL of distilled water, 0.5 μL of a TaqMan™ SNP Genotyping Assay, human (G/T allele, Assay ID: C_8278879_10; ThermoFisher Scientific, Waltham, MA, USA), and 0.5 μL of DNA (20 ng/μL). The reaction was performed in Quant Studio 3 on a default genotyping program (Applied Biosystem, Thermo Fisher Scientific, Waltham, MA, USA). Samples were analyzed in duplicates using a dedicated program: QuantStudio 3/5 Real-Time PCR Software (ThermoFisher Scientific, Waltham, MA, USA). Sample size *n* = 448.

### 2.5. Statistical Analysis

The relative association between allelic/genetic groups was assessed by calculating the odds ratios (ORs) and *p*-values in MedCalc “https://www.medcalc.org/calc/odds_ratio.php (accessed on 14 October 2024)”. ORs, as a measure of the relative risk with 95% confidence intervals (95% CIs), were estimated with logistic regression models and used to compare the allele frequencies in all of the studied groups. The complete haplotype analyses (genotype distribution and chi-square test) were performed on SHEsis online “http://shesisplus.bio-x.cn/SHEsis.html (accessed on 16 October 2024)”. Vitamin 25(OH)D and *VDBP* concentration results are presented as median ± standard deviation (S_D_; GraphPad Software, Inc., La Jolla, CA, USA). Normal distribution was tested using the Shapiro–Wilk test in GraphPad Prism (GraphPad Software, Inc., La Jolla, CA, USA). Comparisons between the tested and control groups were compared using (non-parametric alternative to ANOVA) the Kruskal–Wallis test for three or more groups, where *p* < 0.05 was considered as a significant difference, with Dunn’s multiple comparison test as a post hoc test in GraphPad Prism (GraphPad Software, Inc., La Jolla, CA, USA). A Spearman’s correlation test was performed using GraphPad Prism (GraphPad Software, Inc., La Jolla, CA, USA). Analysis was performed using the group type and coded ordinally to represent health status, where 0 = healthy Control (C), 1 = Control Arthroplasty (CA), and 2 = arthroplasty with Loosening (L). In rs4588/7041, the coding was 0 = TT, 1 = TG, and 2 = GG.

## 3. Results

Between 2019 and 2024 (November to March), 448 Caucasian participants enrolled in this study. The patients were categorized into three groups: (1) 112 individuals from a group of patients with hip replacements in which the transplant was rejected due to loosening (L—Loosening); (2) 87 individuals from a group of patients with functioning hip replacements and no loosening (CA—Control Arthroplasty); and (3) 249 healthy volunteers (C—Control).

### 3.1. Frequencies

Table 2 presents the information on frequencies in the three research groups. For example, among rs7041 in group C, there are 50 subjects with TT (F_TT_ = 0.20; meaning 20%), 144 heterozygous TG (F_TG_ = 0.58; meaning 58%), and 55 subjects with GG (F_GG_ = 0.22, meaning 22%), and in L there are 23 subjects with TT (F_TT_ = 0.20.5, meaning 20.50%), 56 heterozygous TG (F_TG_ = 0.50, meaning 50.1%), and 33 subjects with GG (F_GG_ = 0.294, meaning 29.4%).

### 3.2. Logistic Regression Analysis of rs4588 and rs7041

Logistic regression analysis pointed out that the most common genetic variant in *rs7041* is recessive, which, for every group comparison, L vs. CA +C, L vs. C, and L vs. CA show 2.8-times-higher according to the odds ratio (OR_recessive_ = 2.8) (*p:* <0.0001, 0.0001, and 0.0005, respectively).

The results of the analysis for rs4588 and rs7041 show that, in the groups analyzed, there is a difference between L vs. CA when it comes to rs4588 frequencies (*p* = 0.002). The results show that, in L vs. CA, genotype TT is more frequent than TG with OR_TT vs._ _TG_ = 3.33 and *p* = 0.0007 or than GG with OR_TT vs._ _GG_ = 5.44 and *p* = 0.003. The data are available in Table 2.

Analysis of haplotypes showed that the frequency of G/T is more common in group L than CA + C OR = 1.459, *p* = 0.014, while the G/G haplotype is less frequent (OR = 0.306, *p* = 0.002). In the haplotype analysis, there is also an indication that the G/T haplotype occurs more frequently in group L than in group C (OR = 1.558; 95% CI = 1.135~2.141; *p* = 0.005), while G/G occurs less in group L than in group C (OR = 0.27; 95% CI = 0.121~0.605; *p* = 0.007). The haplotype analysis is presented in the Table 3.

### 3.3. VDBP Concentration

#### 3.3.1. *VDBP* Among the Studied Groups

**Figure 3:**
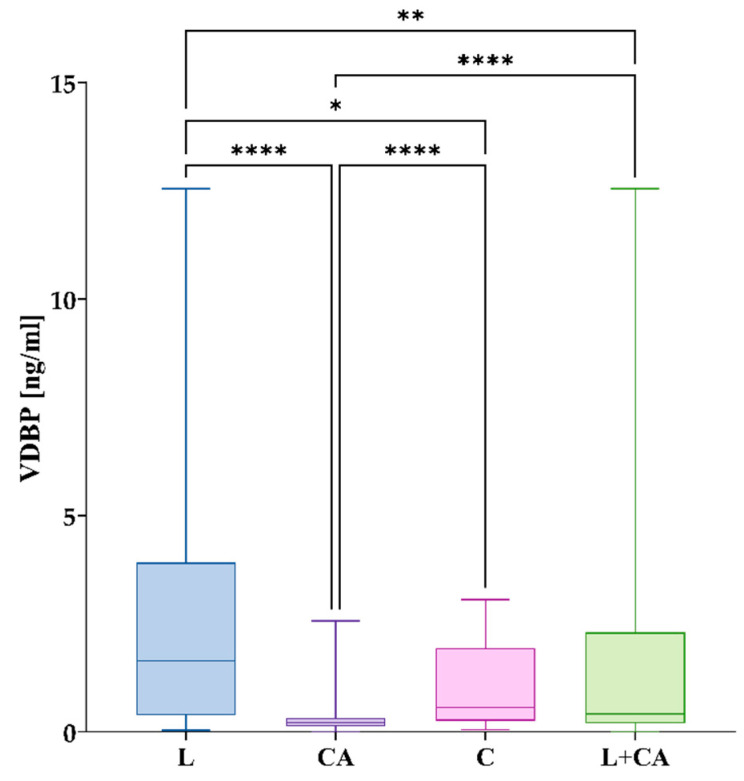
Differences *VDBP* serum concentrations between studied groups. The box plot shows the values in each group studied with whiskers pointing to the min. to max. values, while the lines inside the boxes show medians. Post hoc comparisons after the Kruskal–Wallis test were performed with Dunn’s multiple comparisons test. Figure shows results with *p* < 0.05, where *: *p* < 0.05 (significant at 5%); **: *p* < 0.01 (significant at 1%); ****: *p* < 0.0001 (significant at 0.01%).

In group L, the mean concentration was 2.4 ng/mL, with an S_D_ of 2.6 ng/mL and median of 1.6 ng/mL. In the CA group, the mean was 0.37 ng/mL with an S_D_ of 0.55 ng/mL and median of 0.21 ng/mL. Group C’s mean concentration was 1.1 ng/mL with an S_D_ of 1.0 ng/mL and median of 0.57 ng/mL. There were significant differences between the studied groups (*p* < 0.0001). The differences in *VDBP* concentrations are shown in Figure 3.

#### 3.3.2. *VDBP* Concentrations in Haplotypes Among the Studied Groups

**Figure 4:**
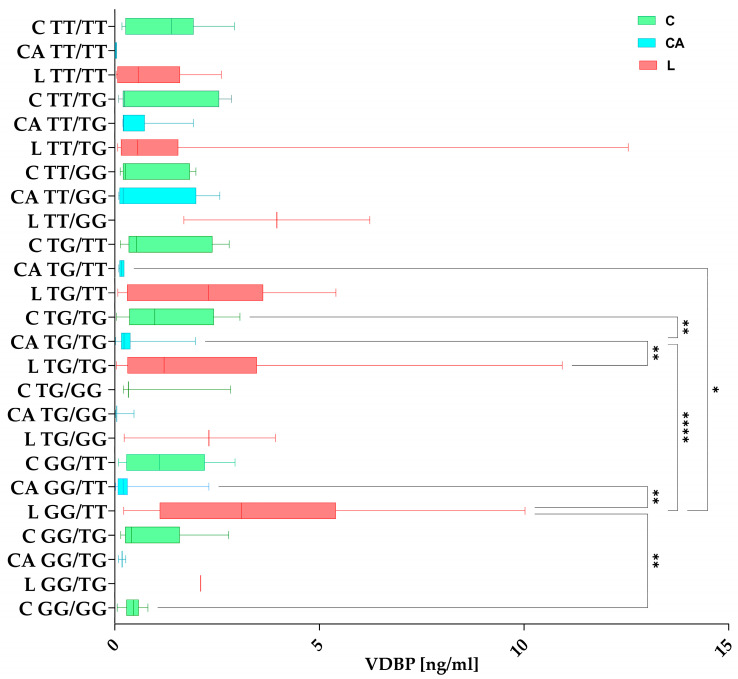
Differences between haplotypes and *VDBP* concentrations among the studied groups (C—Control, green; CA—Control Arthroplasty, blue; L—Loosening, red). The box plot shows the values in each group studied with whiskers pointing to the min. to max. values, while the lines inside the boxes show medians. Post hoc comparisons after Kruskal–Wallis tests were performed with Dunn’s multiple comparisons test. Figure shows results with *p* < 0.05, where *: *p* < 0.05 (significant at 5%); **: *p* < 0.01 (significant at 1%); ****: *p* < 0.0001 (significant at 0.01%).

Groups C, L, and CA were grouped according to mixed rs7041/rs4588 haplotypes and compared for significant differences in the *VDBP* concentration. The differences are shown in Figure 4. The analysis of *VDBP* concentrations was divided across the groups, which is why each haplotype is visible within a given subgroup on the chart. Looking at the top of the chart, the TT/TT designation can be seen for the groups C, CA, and L.

### 3.4. 25(OH)D Concentration

#### 3.4.1. 25(OH)D Among the Studied Groups

**Figure 5:**
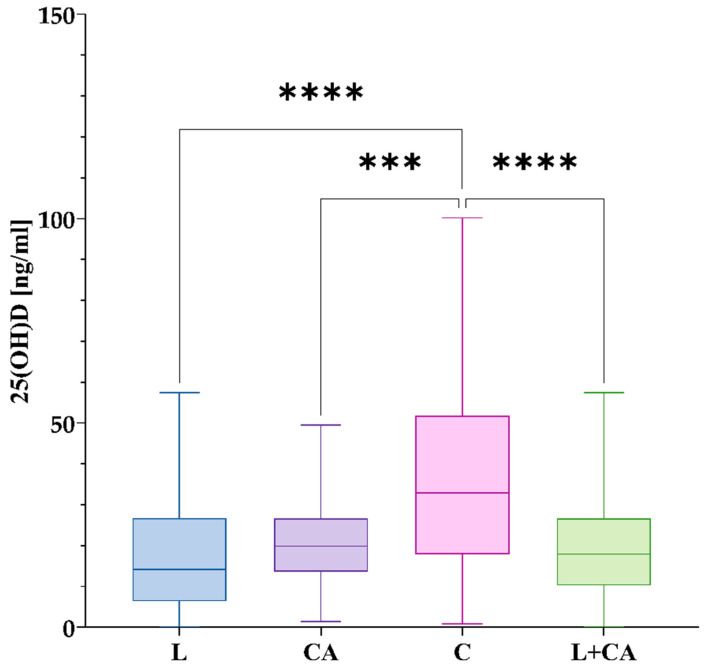
Differences in 25(OH)D concentrations between the studied groups. The box plot shows the values in each group studied with whiskers pointing to min. to max. values, while the lines inside the boxes show medians. Post hoc comparisons after Kruskal–Wallis tests were performed with Dunn’s multiple comparisons test. Figure shows results with *p* < 0.05, where ***: *p* < 0.001 (significant at 0.1%); ****: *p* < 0.0001 (significant at 0.01%).

In group L, the mean concentration was 18.17 ng/mL with an S_D_ of 14.30 ng/mL and a median of 14.16 ng/mL. CA had a higher mean concentration of 20.63 ng/mL with a median of 19.88 ng/mL and S_D_ of 10.11 ng/mL. Group C had the highest mean of 36.44 ng/mL with a median of 32.94 ng/mL and S_D_ of 24.07 ng/mL. Differences among the groups are shown in Figure 5.

#### 3.4.2. 25(OH)D Concentrations in Haplotypes Among the Studied Groups

Groups C, L, and CA were grouped according to mixed rs7041/rs4588 haplotypes and compared for significant differences in the 25(OH)D concentration. For example, rs7041/rs4588 mixed haplotypes are shown separately at the top, and C TT/TT (green) shows the concentration in the control group with the TT/TT haplotype, CA TT/TT (blue) shows the concentration in the Control Arthroplasty group with the TT/haplotype, and L TT/TT (red) shows the concentration in the Loosening group with the TT/TT haplotype.

**Figure 6:**
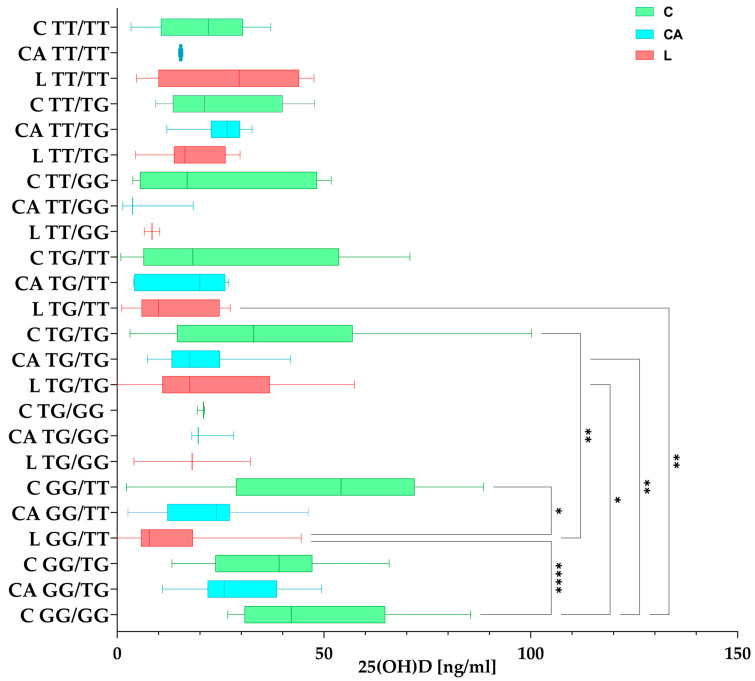
Differences between haplotypes and 25(OH)D concentrations among the studied groups (C—Control, green; CA—Control Arthroplasty, blue; L—Loosening, red). The box plot shows the values in each group studied with whiskers pointing to min. to max. values, while the lines inside the boxes show medians. Post hoc comparisons after Kruskal–Wallis tests were performed with Dunn’s multiple comparisons test. Figure shows results with *p* < 0.05, where *: *p* < 0.05 (significant at 5%); **: *p* < 0.01 (significant at 1%); ****: *p* < 0.0001 (significant at 0.01%).

Differences between haplotypes and 25(OH)D concentration are shown in Figure 6.

### 3.5. Correlation Between Variables

**Figure 7:**
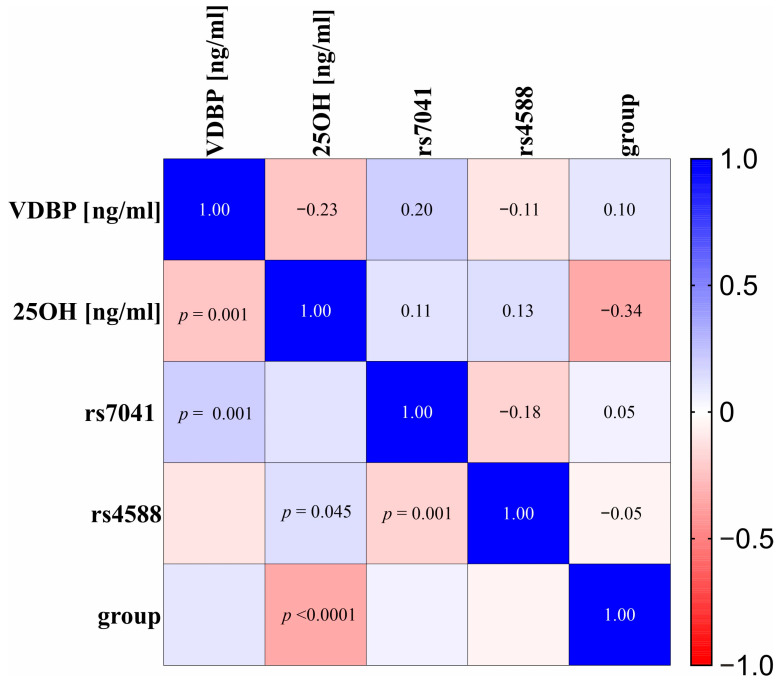
Heatmap of Spearman rank correlation coefficients for associations between both 25(OH)D and *VDBP* serum concentrations, genetic variants of the *VDBP* gene (*rs4588* and *rs7041*), and group type (L, CA, and C) with added *p*-values for significant results. Colors represent the direction and strength of each correlation, where red shades represent negative and blue shades represent positive correlations. Analysis was performed using the group type and coded ordinally to represent health status, where 0 = healthy Control (C), 1 = Control Arthroplasty (CA), and 2 = arthroplasty with Loosening (L). In *rs4588/7041* coding was 0 = TT, 1 = TG, and 2 = GG.

The analysis of correlations showed there is a significant correlation between certain variables. The analysis indicates weak negative relationship between serum levels of 25(OH)D and the VDBP, showing that, as the 25(OH)D levels increase, the levels of the VDBP tend to decrease (R_s_ = −0.23; *p* = 0.001). Another weak but significant correlation was found between VDBP levels and rs7041, where the results suggest a weak positive correlation where patients with the G-allele variant (GG, GT) of the VDBP gene have slightly higher VDBP serum levels compared to the homozygous TT variant (R_s_ = 0.20; *p* = 0.001). A negative correlation was discovered between rs7041 and rs4588, where the data imply that the G-allele in rs7041 is present, and there is a greater likelihood of the T-allele being present in rs4588 (R_s_ = −0.18, *p* = 0.0001). There is also a moderate negative association between the group type (L, CA, and C) and 25(OH)D levels, where 25(OH)D levels tends to increase in the observed CA and L groups (R_s_ = −0.34, *p* < 0.0001) suggesting the protective effects of 25(OH)D among healthy subjects. Statistical significance indicates a weak positive correlation for 25(OH)D and rs4588 G-allele variants (GG, GT) of the VDBP gene, where 25(OH)D levels tend to increase (R_s_ = 0.13; *p* = 0.045). The heatmap of correlations is shown in Figure 7.

## 4. Discussion

This study investigated the role of genetic variations in the *VDBP* gene (rs4588 and rs7041) and serum levels of *VDBP* and 25(OH)D in relation to prosthesis stability and the risk of loosening after a total hip replacement (THR). Consistent with the study’s hypothesis, significant differences in serum concentrations and genotype frequencies were observed among the groups. The L group, associated with prosthesis loosening, exhibited the lowest mean levels of 25(OH)D (18.17 ng/mL) while higher levels of *VDBP* (2.4 ng/mL) compared to higher levels of 25(OH)D were exhibited in the CA (20.63 ng/mL and lower *VDBP*—0.37 ng/mL) and C groups (36.44 ng/mL and 1.1 ng/mL, respectively). Statistical analyses revealed correlations suggesting that higher 25(OH)D levels were linked to lower *VDBP* levels. Additionally, weak positive correlations were identified between rs7041 G allele variants and higher *VDBP* levels, as well as between rs4588 G allele variants and increased 25(OH)D levels. Genotypic analysis highlighted a recessive model for rs7041 and a dominant model for rs4588 as significant predictors. Haplotype analysis showed that the G/T haplotype was more frequent in the L group, while the G/G haplotype was less common. These findings indicate that specific genetic variants and altered serum levels of 25(OH)D and *VDBP* may contribute to prosthesis instability, supporting this study’s objective of identifying biochemical and genetic factors associated with this complication.

A review of the literature shows that only a few studies have investigated vitamin D in the context of total hip replacement (THR) or total knee arthroplasty (TKA). Moreover, most researchers have focused exclusively on the measurement of vitamin D (25(OH)D) levels in preoperative or postoperative groups [41,42,43,44]. There are not many publications on other vitamin D metabolic pathway components, which prompts us to continue our research on THR patients and VD metabolism, including other factors, such as receptors [28] or the major transport protein—the *VDBP*.

Vitamin D serum concentration not only depends on diet, supplementation, or the effects of UVB radiation on the skin, but also on genetic factors. One of these is SNPs, single nucleotide polymorphisms, which can influence gene functions, such as phenotypic variation. The assignment of functions to SNPs is necessary to advance the development of preventive medicine [45]. SNPs can change coded amino acids (nonsynonymous) [46], remain silent (synonymous) [47], or occur in non-coding regions. They can influence promoter activity (gene expression), the conformation of messenger RNA (mRNA) (affecting its stability, structure, and protein folding), and the subcellular localization of mRNAs or proteins. These changes can affect cellular processes and the cellular response to therapeutic targets, functions of proteins, and potentially contribute to disease development [47,48]. There are many recent studies linking the effects of *VDBP* polymorphisms to *VDBP* and/or vitamin D status or *VDBP* status to vitamin D status [35,49,50,51,52,53,54,55]. There are studies on vitamin D status among total hip or total knee arthroplasty patients [41], however these studies do not include *VDBP* levels or its gene variations. We would like to extend this knowledge to include the structures of *VDBPs* that are essential for proper metabolism in the body—not only vitamin D, but also the transporter protein.

We recently analyzed SNPs in the *VDR* gene and its correlation with 25(OH)D serum levels [28]. The results have prompted us to expand our research on other proteins involved in the VD pathway, such as the *VDBP*.

Vitamin D is a nutrient that plays a great role in the calcium and phosphorous homeostasis and maintenance of muscle mass and bone health [56]. In addition, higher VD levels have been linked to a reduced risk of rickets and various autoimmune, infectious, and allergic diseases [57]. The *VDBP* as a VD transporter is a key component that binds and transports VD metabolites to the liver and kidneys or other organs where they are activated through hydroxylations [26,58,59].

Da Cunha et al. investigated the association between 25(OH)D levels and outcomes in 110 patients undergoing a total hip replacement. They found a weak correlation between 25(OH)D levels and changes in peak strength and peak power. The authors hypothesized that patients with higher 25(OH)D levels experienced better muscle recovery after a THR [60]. This could confirm the role of vitamin D in achieving positive outcomes after surgery. Our results support the hypothesis that median 25(OH)D serum concentration levels are higher in the Control Arthroplasty group, where no loosening occurred. We want to highlight the differences in the sample testing, as our results were obtained using ELISA on serum, whereas de Cunha et al. used peripheral venous blood samples analyzed with liquid chromatography coupled with tandem mass spectrometry and included only 110 subjects.

The prevalence of a low vitamin D status in patients undergoing a THR was determined by Unnanuntana et al. [61] in a group of 200 patients, including 88 men and 112 women. Preoperative diagnoses were OA (187 patients), osteonecrosis (six patients), development of hip dysplasia (three patients), and post-traumatic OA (four patients). Approximately 40% of patients in Unnanantana et al.’s study was found to have low serum vitamin D levels. Our findings support this, as orthopedic patients in the L and CA groups exhibited lower serum vitamin D levels compared to the healthy subjects. Unnanuntana used a laboratory radioreceptor assay in contrast to our ELISA. However, overall results suggest that the orthopedic patient group (L + CA) has an overall lower vitamin D status than the non-orthopedic group, emphasizing the critical role of vitamin D in maintaining a healthy bone status. This is further supported by the findings of Hussain et al. [62] whose research on a cohort of 9235 participants demonstrated a significant correlation between higher serum 25(OH)D concentrations and an increased risk of hip arthroplasty for osteoarthritis (OA) in men. The authors stated that the underlying mechanisms linking serum 25(OH)D concentrations and the risk of hip OA requiring hip arthroplasty remain unclear. However, it has been suggested that increased bone mineral density (BMD) is also associated with vitamin D status and its regulatory effects on the bone formation process [62]. Hussain et al. used a direct competitive chemiluminescent immunoassay [62], which is a different and more sensitive technique. However, it is similar to other immunoassays, as both rely on antigen–antibody binding specificity to detect the presence or concentration of a target molecule. Bone health is a complex issue that also involves sex hormones. The differences in 25(OH)D levels and the risk of hip arthroplasty outcomes between men and women might occur due to sex-specific differences. With aging, the pathophysiology of bone loss differs between males and females. In elderly women, there is an increased rate of bone remodeling with a negative remodeling balance, where bone resorption exceeds formation, leading to bone loss and the disruption of bone microarchitecture. In elderly men, aging primarily results in reduced bone formation and low bone turnover [63,64,65]. Our studies did not support the differences in THR outcomes according to gender and showed the opposite result for 25(OH)D action, indicating a moderate negative association between group type (L, CA, and C) and 25(OH)D levels, with 25(OH)D levels tending to be higher in non-orthopedic groups. This suggests a protective effect of 25(OH)D in healthy individuals. In a study conducted by Maier et al. involving a total of 1083 patients scheduled for elective knee or hip arthroplasty, it was found that serum vitamin D levels were inversely related to the length of stay in the orthopedic department when compared to patients with normal vitamin D levels. In multivariate analyses, the length of stay remained significantly associated with 25(OH)D levels [66]. The results of 25(OH)D measurements are based on chemiluminescent microparticle immunoassay (CMIA) technology, which also differs from standardized ELISA. These results support our findings, where most orthopedic patients exhibit significant 25(OH)D deficiencies. Our data show a moderate inverse relationship between vitamin D levels and group type, with lower vitamin D concentrations observed in patients with prosthesis loosening compared to healthy individuals and those with prostheses without loosening. This could contribute to poor THR or TKA outcomes in the future, highlighting the importance of vitamin D status for bone health.

Kim Hong Seok et al. published a study comparing vitamin D deficiency in elderly patients undergoing hip fracture surgery (HFS) and elective primary total hip replacement (THR). The study included 70 HFS patients (42 women and 28 men) and 100 THR patients (74 women and 26 men). The results showed lower vitamin D levels in THR patients compared to the HFS group. In the HFS group, vitamin D deficiency was more pronounced in sarcopenic patients, with an insufficiency found in 80% of HFS patients and 36% of THR patients [62]. Our results support these findings and point to low serum vitamin D level as a predictive factor of prostheses loosening. It is important to note that Kim Hong Seok et al. used a radioactive isotope (I-125) to measure 25(OH)D, which differs from ELISA.

Another study by Traven et al., which aligns with our findings, demonstrates that lower vitamin D levels are associated with an increased risk of complications following orthopedic surgery. A retrospective review of 126 revision THR patients found that low vitamin D levels were not linked to a 30-day re-admission risk, but were associated with increased 90-day complications, including periprosthetic joint infections. Vitamin D deficiency was more prevalent in the TJA revision population (55%) compared to the general population (8%). Patients with low vitamin D had a higher rate of postoperative complications (20.3%) than those with normal levels (8.8%, *p* < 0.001) [67]. The authors did not disclose which method was used to determine 25(OH)D concentrations.

On the other hand, our results are not supported by Zajonz et al., who studied the significance of vitamin D in periprosthetic infections after a THR and TKA. They found no significant differences in 25(OH)D levels between the groups, although there was a difference in the calcium levels [68]. Similar studies were published by Visser et al. who conducted a study involving 87 patients scheduled for a THA. Of these, 23 were classified as vitamin D deficient, 32 as insufficient, and 32 as sufficient. The authors found no relationship between pre-surgery vitamin D status and post-surgery physical performance, potentially due to seasonal fluctuations in vitamin D levels during sample collection (September to March). The study does not explicitly mention the method used for detecting 25(OH)D levels [69].

To our knowledge, there is no study linking susceptibility to THR to *VDBP* serum levels or genetic variability, such as *rs4588* or *rs7041*. However, there are some studies linking *VDBP* and its genetic variants to bone-related diseases, bone mass, or bone formation. Studies investigating the effects of *VDBP* on bone health are mostly limited to bone metabolism and focus on osteoporosis as a heterogeneous group of bone disease characterized by a low skeletal mass.

Low *VDBP* levels as a part of vitamin D metabolism have been associated with low BMD and could be considered as a novel, non-invasive biomarker for the early detection of osteoporosis. As *VDBP* levels were significantly lower in the postmenopausal women group, it could potentially be a simple biomarker for osteopenia and osteoporosis [70].

The researchers not only focus on *VDBP* levels, but also on investigating the major genetic forms of *VDBP* variants, as they show differences in affinity for 25(OH)D and 1,25(OH)_2_D. According to Bhan, GC2 (Asp/Lys) has the lowest affinity to 25(OH)D, GC1S has an intermediate affinity, and GC1F has the highest affinity to 25(OH)D. In summary, the combination of various alleles may influence not only the levels, but *VDBP* action on target tissues, including bones [71].

The study of Takiar et al. did not provide conclusive data on the *VDBP* and the association of *rs4588* or *rs7041* with fracture risk, although 25(OH)D deficiency levels were associated with a higher incidence of hospitalized fractures [72]. As the vitamin D deficiency results support our studies, we find that genetic factors of rs4588 and rs7041 in the *VDBP* may be predictive, as our results reveal. Specifically, the analysis of rs4588 and rs7041 revealed significant associations in the studied groups. For rs4588, there was a notable difference in allele frequencies between the L and CA groups (*p* = 0.002). Contingency analysis further demonstrated overall associations between genetic variants (genotypes) and implant loosening in the L group compared to CA + C (χ^2^ = 7.565, *p* = 0.005) and L compared to C (χ^2^ = 16.87, *p* = 7.51 × 10^−4^).

Logistic regression analysis for rs4588 indicated a higher likelihood in L vs. CA (OR_dominant_ = 2.42, *p* = 0.02). Additionally, the TT genotype was significantly more common in L vs. CA compared to TG (OR_TT_ vs. _TG_ = 3.33, *p* = 0.0007) or GG (OR_TT_ vs. _GG_ = 5.44, *p* = 0.003). These genetic findings, coupled with the observed associations between vitamin D deficiency and the outcomes, support the role of both genetic and vitamin D-related factors in influencing postoperative complications.

Li et al. found that VDBP levels and genetic variants are associated with 25(OH)D, which is also confirmed by our studies. The TT variant of rs7041 was more frequent (0.543) than TG (0.370) and GG (0.74), and rs4588 GT was as frequent (0.436) as GG (0.446), but TT had the lowest frequency (0.118) among the studied postmenopausal women [73].

A summary of all the findings above is presented in Table 4.

The limitation of this study is the potential imbalance of sample sizes across groups, which could introduce bias and reduce the precision of statistical comparisons. If the sample size in one group is much smaller than in others, it may result in the overestimation or underestimation of effects, particularly in smaller or less representative groups, like the L group. However, frequency calculations did not present that risk.

Another limitation is the reliance on serum measurements of 25(OH)D, which can be influenced by various factors, such as time of year (this is why we limited the serum measurements to autumn/winter), diet, or recent supplementation, leading to potential variability in and the imprecision of these measurements. The lack of longitudinal data also limits the ability to track changes over time and assess the long-term impact of genetic and biochemical factors on prosthesis stability. Furthermore, genetic factors other than the studied SNPs (*rs7041* and *rs4588*) may also play a role in prosthesis loosening but were not considered, which could lead to residual confounding. Lastly, selection bias could arise if patients with prosthesis loosening are not representative of the general population or if they differ systematically from patients in other groups, affecting the external validity of the findings. While this study found significant associations, these effects may be influenced by uncontrolled variables, limiting the generalizability of the results to broader populations.

The statistical analysis provided shows the relationship between variables, but it is important to note that the Spearman rank correlation measures the strength and direction of a monotonic relationship between two variables. While it can identify statistically significant correlations, even weak correlations may achieve significance in large sample sizes, which does not imply a strong predictive value.

The results from similar studies should be considered, as they can help validate the findings or highlight potential discrepancies. While some studies have suggested associations between vitamin D levels and bone health, and genetic variations influencing prosthesis outcomes, the findings in this study should be viewed cautiously, given the multiplicity of analyses conducted.

Overall, while this study presents promising associations between genetic and biochemical factors and prosthesis stability, further research, especially longitudinal studies with larger, more balanced sample sizes, is needed to confirm these findings and determine their clinical relevance. Additionally, addressing other potential confounding factors and exploring other genetic variations related to prosthesis loosening would be important for further refining our understanding of the mechanisms involved.

This study’s focus on a specific complication of THR—prosthesis loosening—means that the results may not be applicable to other complications or outcomes related to THR, such as infection or revision surgery. This narrow focus reduces the broader applicability of the findings to the overall patient population undergoing hip replacement surgeries.

## 5. Conclusions

Diagnostic tests usually measure 25(OH)D, the primary inactive metabolite of vitamin D. However, it is crucial to also consider other components involved in the metabolism and transport of vitamin D (as shown in Figure 1) and to include additional parameters that could be associated with pathological conditions in the body. Furthermore, a simple and informative approach could be to identify SNP mutations in the *VDBP* gene that may indicate a predisposition to VD deficiency. Once a SNP is identified, it remains unchanged for life. The negative correlation between *VDBP* and 25(OH)D associated with higher *VDBP* levels in orthopedic patients indicates that an adaptive or compensating mechanism may be present in this group in response to a lower vitamin D status. The correlation between vitamin D and *VDBP* levels with prosthesis loosening could help identify high-risk patients. By assessing both genetic and biochemical markers preoperatively, clinicians could stratify patients based on their risk of implant instability and tailor their follow-up care accordingly. This could involve more frequent monitoring for signs of loosening or earlier intervention if necessary.

## Figures and Tables

**Table 1:** Characteristics of groups.

Characteristics	L (*n* = 112)	CA (*n* = 87)	C (*n* = 249)	*p*-Value (L vs. CA; L vs. C; CA vs. C)
Age: years mean (SD)	53.57 (12.37)	48.77 (14.52)	44.53 (13.23)	0.0156/<0.0001/0.344 *
Gender: *n* (%)				
Female:	50 (45)	25 (29)	122 (49)	
Male:	62 (55)	62 (71)	127 (51)	
		χ^2^ (2, 448) = 10.7693		0.0046

* non parametric Anova: Kruskal-Wallis.

**Table 2:** Genotype frequencies with logistic regression analysis of the dominant and recessive models of rs7041 and rs4588 in *VDBP* gene polymorphisms and χ^2^ in the studied groups and the associations with prosthesis loosening.

	*rs7041*			*rs4588*		
Genotype	Control [*n* (%)]	Control Arthroplasty [*n* (%)]	Loosening [*n* (%)]	Control [*n* (%)]	Control Arthroplasty [*n* (%)]	Loosening [*n* (%)]
	C	CA	L	C	CA	L
TT	50 (20)	15 (17.2)	23 (20.5)	119 (47.7)	60 (68.9)	51 (45.5)
TG	144 (58)	52 (59.7)	56 (50.1)	39 (15.6)	8 (9.1)	12 (10.7)
GG	55 (22)	20 (22.9)	33 (29.4)	91 (36.5)	19 (21.8)	49 (43.7)
	Genotype χ^2^ (4, 448) = 0.3205; *p* = 0.5242			Genotype χ^2^ (4, 448) = 16.14; *p* = 0.0028		
	**L vs. CA + C**	**L vs. C**	**L vs. CA**	**L vs. CA + C**	**L vs. C**	**L vs. CA**
Dominant OR	0.68	0.68	0.72	1.36	1.55	2.49
(95% CI; *p*)	(0.43–1.11; 0.12)	(0.41–1.12; 0.13)	(0.38–1.36; 0.31)	(0.69–2.67; 0.38)	(0.78–3.08; 0.21)	(1.14–5.42; 0.02)
Recessive OR	2.8	2.8	2.8	1.02	0.97	0.93
(95% CI; *p*)	(1.71–4.59; <0.0001)	(1.69–4.65; 0.0001)	(1.56–5.00; 0.0005)	(0.67–1.55; 0.93)	(0.62–1.51; 0.87)	(0.55–1.56; 0.78)
TT OR	1 (reference)					
vs. TG OR	1.23	1.18	1.42	1.57	1.26	3.33
(95% CI; *p*)	(0.71–2.17; 0.46)	(0.66–2.12; 0.57)	(0.67–3.02; 0.36)	(0.99–2.47; 0.06)	(0.78–2.03; 0.34)	(1.66–6.68; 0.0007)
vs. GG OR	0.80	0.77	0.93	1.75	1.75	5.44
(95% CI; *p*)	(0.43–1.51; 0.50)	(0.40–1.48; 0.43)	(0.40–2.19; 0.87)	(0.85–3.58; 0.13)	(0.83–3.65; 0.14)	(2.17–13.67; 0.0003)

**Table 3:** Haplotype analyses of rs7041 and 4588 in L vs. CA + C, L vs. CA, and L vs. C, with genotype frequencies and OR.

L vs. CA + C Haplotype	Loosening (Freq)	Control Arthroplasty + Control (Freq)	χ^2^	Fisher’s *p*	Pearson’s *p*	OR [95% CI]
rs7041/4588						
TT	34 (0.151)	117 (0.174)	0.597	0.472	0.439	0.848 [0.56–1.286]
TG	68 (0.303)	209 (0.311)	0.043	0.867	0.834	0.965 [0.695–1.34]
GT	115 (0.513)	282 (0.419)	5.983	0.016	0.014	1.459 [1.077–1.976]
GG	7 (0.031)	64 (0.095)	9.427	0.001	0.002	0.306 [0.138–0.678]
**L vs. CA haplotype**	**Loosening (freq)**	**Control Arthroplasty (freq)**	**χ^2^**	**Fisher’s *p***	**Pearson’s *p***	**OR [95% CI]**
rs7041/4588						
TT	34 (0.151)	17 (0.097)	2.564	0.13	0.109	1.652 [0.889–3.07]
TG	68 (0.303)	65 (0.373)	2.156	0.163	0.142	0.73 [0.48–1.111]
GT	115 (0.513)	81 (0.465)	0.898	0.364	0.343	1.211 [0.814–1.801]
GG	7 (0.031)	11 (0.063)	2.317	0.148	0.127	0.478 [0.181–1.259]
**L vs. C** **haplotype**	**Loosening (freq)**	**Control (freq)**	**χ^2^**	**Fisher’s *p***	**Pearson’s *p***	**OR [95% CI]**
rs7041/4588						
TT	34 (0.151)	100 (0.2)	2.456	0.121	0.117	0.712 [0.465–1.09]
TG	68 (0.303)	144 (0.289)	0.154	0.724	0.694	1.071 [0.759–1.512]
GT	115 (0.513)	201 (0.403)	7.565	0.007	0.005	1.558 [1.135–2.141]
GG	7 (0.031)	53 (0.106)	11.459	0.0004	0.0007	0.27 [0.121–0.605]

Haplotypes with a frequency < 0.03 are ignored.

**Table 4:** Summary table of the correlations and significant differences in *VDBPs* or 25(OH)D among cases on bone-related diseases or procedures (THA/TKA).

Results	Study Cohort	Case	References
	25(OH)D Conc.		
Weak correlation between 25(OH)D levels and peak stretch or peak power	110 subjects	THR	[60]
23 vitamin D-deficient, 32 vitamin D-insufficient, and 32 vitamin D-sufficient patients; 25(OH)D status not related to performance after surgery	87 subjects	THR	[69]
40% of patients were noted to have low 25(OH)D levels	200 subjects; 187—osteoarthritis (OA), 6—osteonecrosis, 3—hip dysplasia, and post-traumatic OA—4	THR	[74]
Correlation of increased 25(OH)D serum concentrations and increased risk of total hip replacements in OA among males, but not among females	9135 subjects	THR, BMD	[62]
Insufficient serum levels of 25-OH-D were observed in 86% of patients, with over 60% classified as vitamin D deficient	1083 subjects	THR/TKA	[66]
25(OH)D levels lower among patients for THR n for HFS	70 subjects for HFS; 100 subjects for THR	HFS, primary THR	[75]
No association of 25(OH)D with a re-admission risk, but elevated risk of 90-day complications and periprosthetic joint infections	126 subjects	revision THR	[67]
No significant differences in 25(OH)D and studied groups	240 subjects	THR/TKA, osteoporosis	[68]
Deficient levels of 25(OH)D associated with increased fracture risk. Racial differences in fracture risk may be related to differences in bioavailable vitamin D due to the single nucleotide polymorphism	12,781 middle-aged subjects	fracture risk	[72]
	**VDBP conc.**		
Low VDBP serum levels are associated with a low BMD	425 postmenopausal women	BMD	[70]
	**VDBP SNP**		
TT variants of rs7041 were more frequent (0.543) than TG (0.370) and GG (0.74) and rs4588 GT was as frequent (0.436) as GG (0.446), but TT had the lowest frequency (0.118) rs4588 has no association with blood VDBP	967 postmenopausal women	BMD	[73]
No independent association of rs4588 and rs7041 with fractures	12,781 middle-aged subjects	fracture risk	[72]

## Data Availability

The authors confirm that the data supporting the findings of this study are available within the article. Correspondence and requests for materials should be addressed to D.R.
